# Methylidynetrisphosphonates: Promising C_1_ building block for the design of phosphate mimetics

**DOI:** 10.3762/bjoc.9.114

**Published:** 2013-05-24

**Authors:** Vadim D Romanenko, Valery P Kukhar

**Affiliations:** 1 Institute of Bioorganic Chemistry and Petrochemistry, National Academy of Sciences, 02660 Kyiv, Ukraine. Fax: 38 044 5732552; Tel: 38 044 5732525

**Keywords:** biomimetic synthesis, C_1_ building blocks, phosphorylation, polyphosphonates, synthetic methods

## Abstract

Methylidynetrisphosphonates are representatives of geminal polyphosphonates bearing three phosphonate (PO_3_H_2_) groups at the bridged carbon atom. Like well-known methylenebisphosphonates (BPs), they are characterized by a P–C–P backbone structure and are chemically stable mimetics of the endogenous metabolites, i.e., inorganic pyrophosphates (PP_i_). Because of its analogy to PP_i_ and an ability to chelate metal ions, the 1,1,1-trisphosphonate structure is of great potential as a C_1_ building block for the design of phosphate mimetics. The purpose of this review is to present a concise summary of the state of the art in trisphosphonate chemistry with particular emphasis on the synthesis, structure, reactions, and potential medicinal applications of these compounds.

## Introduction

Methylidynetrisphosphonic acid, HC(PO_3_H_2_)_3_, or more commonly methylidynetrisphosphonates, XC(PO_3_R_2_)_3_, also called methanetrisphosphonates, are representatives of geminal polyphosphonates among which methylenebisphosphonates, H_2_C(PO_3_R_2_)_2_, are well-known as metabolically stable analogues of the naturally occurring inorganic pyrophosphate (PP_i_) [1–3]. Bisphosphonates are widely used drugs for the treatment and prevention of excessive osteoclast-mediated bone resorption associated with osteoporosis, Paget's disease, and tumour-induced osteolysis [4–6]. In the wide area of polyphosphonate chemistry, *gem*-trisphosphonates represent a new area of study and many of the important developments in their chemistry are based on their specific properties. The presence of three phosphonate (PO_3_R_2_) substituents at the bridged carbon atom causes pronounced physical and chemical effects and imparts unique electronic characteristics to 1,1,1-trisphosphonylated compounds. Most importantly, the replacement of the hydrogen atom attached to the bridge carbon in methylenebisphosphonates by a third ionisable phosphonate moiety results in *supercharged isosteric systems* relative to pyrophosphoric acid [7]. It was also demonstrated that steric effects play a significant role in trisphosphonate chemistry and allow efficient control of regio- and stereochemistry for addition reactions. Moreover, examples of the reactivity of functionalized methylidynetrisphosphonates XC(PO_3_R_2_)_3_ shown in this review indicate that the nature of the substituent X at the central carbon atom is the key to the optimization of their acidic and coordination properties thus providing excellent possibilities for the design of effective phosphate mimetics. Especially successful so far seem to be approaches for the synthesis of trisphosphonate-modified nucleotides and nucleosides, which represent a promising class of potential drugs [8].

## Review

### Preparation of methylidynetrisphosphonates

#### Synthesis via a Michaelis–Arbuzov reaction

**Scheme 1:**
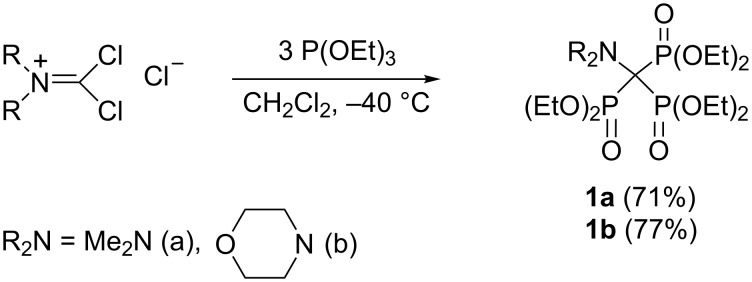
Synthesis of hexaethyl dialkylaminomethylidynetrisphosphonates **1** from dichloromethylene dialkylammonium chlorides.

**Scheme 2:**
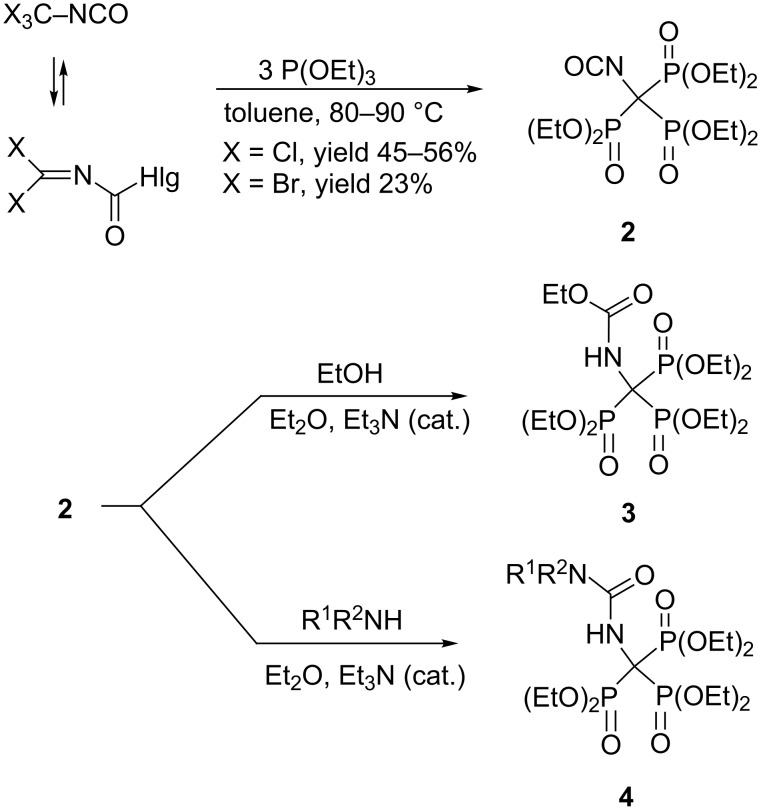
Synthesis and some transformations of trisphosphonate **2**.

Phosphonate esters are often prepared through Michaelis–Arbuzov reaction of a trialkyl phosphite with an alkyl halide [9]. However, contrary to the early patent claims, trisphosphonate esters cannot be derived from simple trihalomethyl derivatives [10–12]. Indeed, chloroform and bromoform do not react with triethyl phosphite even under harsh conditions [11]. In addition, no reaction occurred between benzotrichloride (PhCCl_3_) and triethyl phosphite at a temperature below 150 °C. The CuCl-catalyzed reaction of PhCCl_3_ and (EtO)_3_P at 120–140 °C produced 1,2-diphenyl-1,1,2,2-tetrachloroethane [13]. The early work reported that the trisphosphonate PhC(PO_3_Et_2_)_3_ was formed from triethyl phosphite and dibenzoyl peroxide when reagents were boiled under reflux in chloroform, but proof of the trisphosphonate structure was not given [14]. It was later suggested that the product of this reaction is most likely a phosphate-phosphonate, PhC(PO_3_Et_2_)_2_OPO_3_Et_2_ [15]. Birum postulated the preparation of the trisphosphonate (Et_2_O_3_P)_3_CSPO_3_Et_2_ from Cl_3_CSCl and P(OEt)_3_, but once again, no definitive proof of trisphosphonate structure was provided [16–17]. The first authentic synthesis of 1,1,1-trisphosphonate derivatives **1a**,**b** was described by Kukhar, Pasternak and Kirsanov by allowing the addition of triethyl phosphite to dichloromethylene dialkylammonium chloride to proceed in dichloromethane at −40 °C (Scheme 1) [18]. Other researchers have confirmed this finding [19–20]. Trichloromethylisocyanate [21] and tribromomethylisocyanate [22] react similarly with 3 equivalents of triethyl phosphite forming isocyanate **2,** which was characterized by its IR and NMR spectra and also by its reaction products **3** and **4** (Scheme 2). Trisphosphonate **2** is also formed by the reaction of [(EtO)_2_P(O)]_2_C(Cl)NCO as well as (EtO)_2_P(O)CCl_2_NCO with 1 or 2 equiv of (EtO)_3_P, correspondingly [21].

**Scheme 3:**
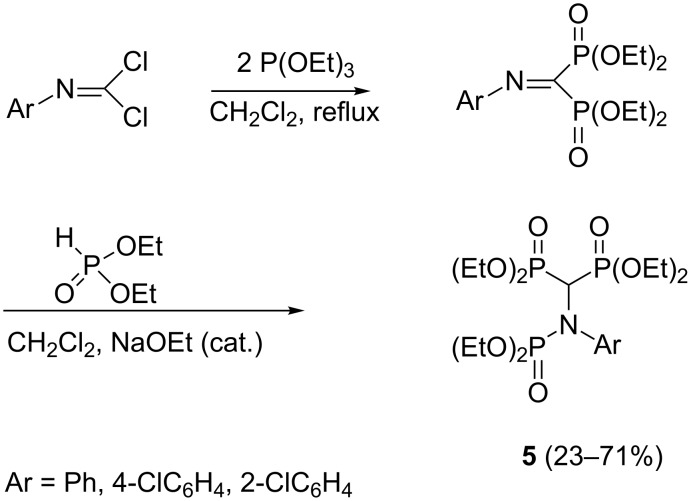
Attempt to synthesize trisphosphonates by the combination of Arbuzov reaction and dialkyl phosphite addition.

An unsuccessful attempt to synthesize trisphosphonates from isocyanide dichlorides was made by the combination of Arbuzov reaction and dialkyl phosphite addition. The reaction sequence leads to *N*-phosphorylated bisphosphonates **5** instead of the desired trisphosphonates (Scheme 3) [23–24].

#### Synthesis via anionic methylenebisphosphonates

**Scheme 4:**
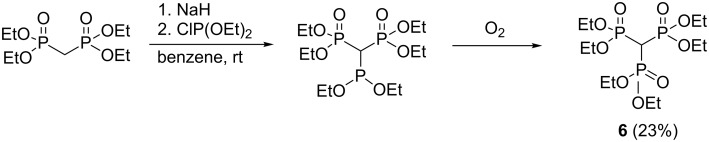
Synthesis of hexaethylmethylidynetrisphosphonate **6** via phosphinylation of tetraethyl methylenebisphosphonate anion.

**Scheme 5:**
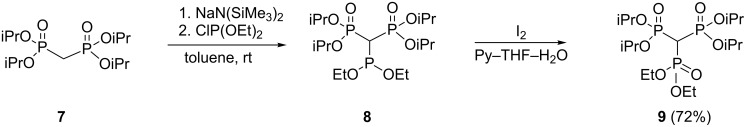
Synthetic approach to methylidynetrisphosphonate ester **9**.

The anionic bisphosphonate [CH(PO_3_Et_2_)_2_]^−^ proved unreactive with diethyl chlorophosphate, ClP(O)(OEt_2_)_2_, but phosphinylation of the bisphosphonate anion with diethyl chlorophosphite, ClP(OEt)_2_, followed by in situ oxidation with atmospheric oxygen of the presumed phosphinate intermediate to the trisphosphonate **6** was successful (Scheme 4) [25]. An improved version of this method includes oxidation of the phosphinate intermediate with hydrogen peroxide in tetrahydrofuran [26]. Mixed ethyl/isopropyl trisphosphonate ester **9** has been prepared by treatment of the bisphosphonate **7** with diethyl chlorophosphite and sodium hexamethyldisilazane, and subsequent oxidation of the phosphinate intermediate **8** with iodine in pyridine–THF–H_2_O (Scheme 5) [7,27]. It was noted that the intermediate **8** was unstable during either acidic or basic workup and readily decomposed to the starting bisphosphonate **7**. However, oxidation of **8** gave stable trisphosphonate ester **9** in 72% yield.

**Scheme 6:**
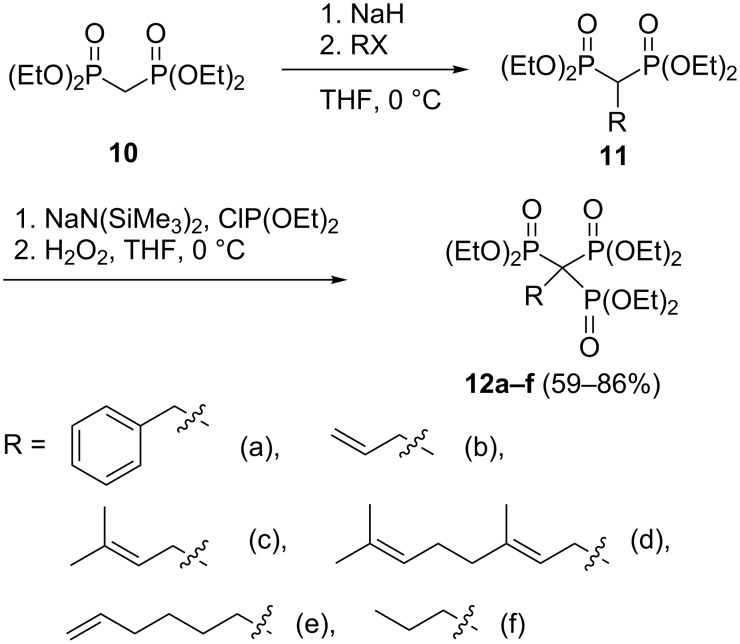
Synthesis of alkylidyne-1,1,1-trisphosphonate esters **12**.

**Scheme 7:**
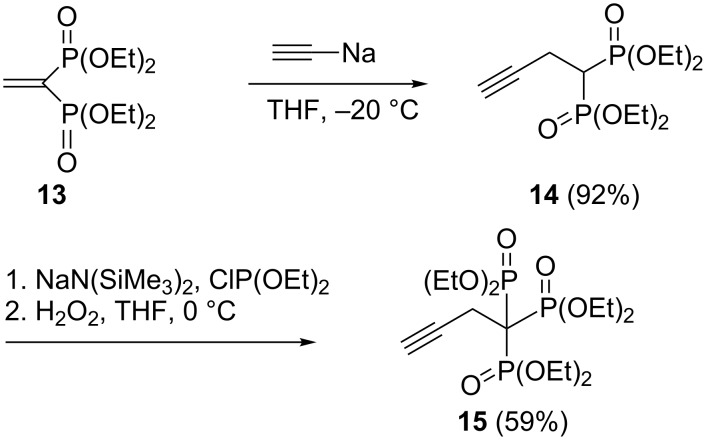
Two-step one-pot synthesis of propargyl-substituted trisphosphonate **15**.

For the synthesis of alkylidyne-1,1,1-trisphosphonate esters **12a**–**f** a three-step protocol including synthesis of alkylidene-1,1-bisphosphonates **11** from tetraethyl methylenebisphosphonate (**10**), phosphinylation and oxidation has been developed (Scheme 6) [26]. Propargyl-substituted trisphosphonate **15** was prepared by conjugate addition of sodium acetylide to ethenylidenebisphosphonate **13** and the subsequent phosphinylation and oxidation (Scheme 7). This compound has been used in an elegant construction (“click” chemistry) of triazole derivatives (see Scheme 17) [26,28].

#### Synthesis via 7,7-bisphosphonoquinone methides

**Scheme 8:**
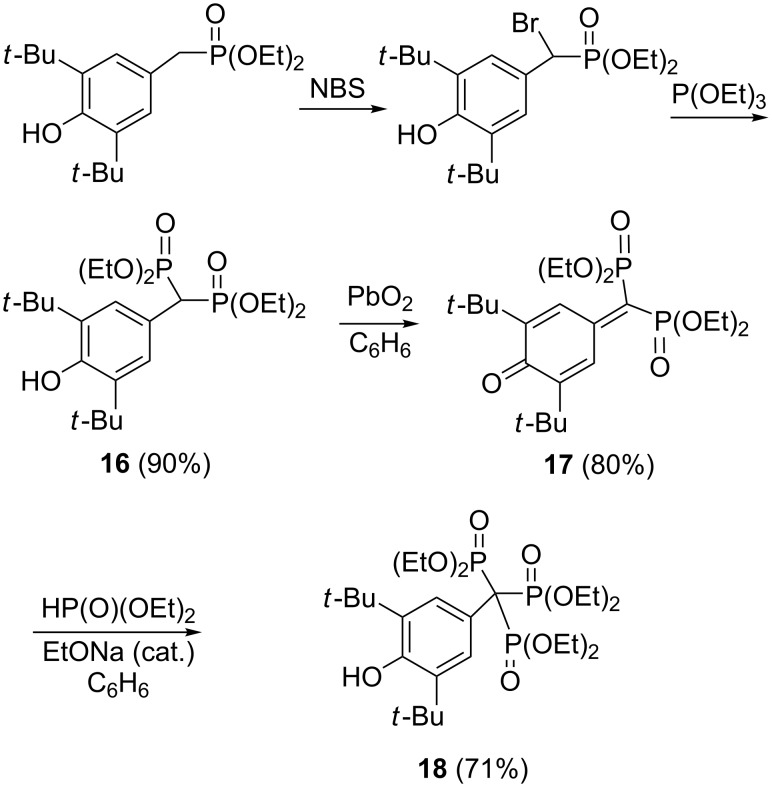
Synthetic route to trisphosphonate **18** via 7,7-bisphosphonyl-3,5-di-*ter*t-butylquinone methide **17**.

**Scheme 9:**
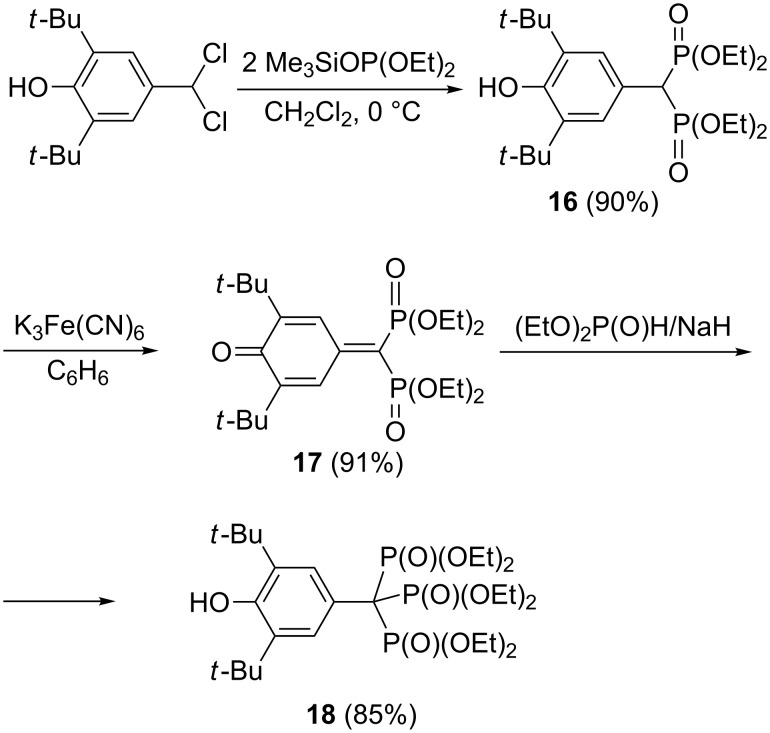
Synthesis of trisphosphonate **18** starting from 2,6-di-*tert*-butyl-4-(dichloromethyl)phenol.

A unique route to trisphosphonate **18** via addition of diethyl phosphite to 7,7-bisphosphonyl-3,5-di-*tert*-butylquinone methide **17** derived by oxidation of bisphosphonate **16** was developed by Gross and co-workers (Scheme 8) [29]. More recent reports of this type of reaction are from laboratories of Russian researchers [30–31]. They showed that bisphosphonate **16** can be prepared in good yield by the Arbuzov reaction of trimethylsilyl esters of trivalent phosphorus acids with the easily accessible 2,6-di-*tert*-butyl-4-(dichloromethyl)phenol. In the next step, the bisphosphonate **16** was oxidized with K_3_Fe(CN)_6_ into quinone methide **17** in 91% yield. Further addition of diethyl phosphite in the presence of sodium hydride gives the triphosphonate **18** (Scheme 9). Note that bisphosphonate **16** is also available by the reaction 4-hydroxy-3,5-di-*tert*-butylbenzaldehyde with triethyl phosphite in the presence of malonic ester (yield 53%) [32].

**Scheme 10:**
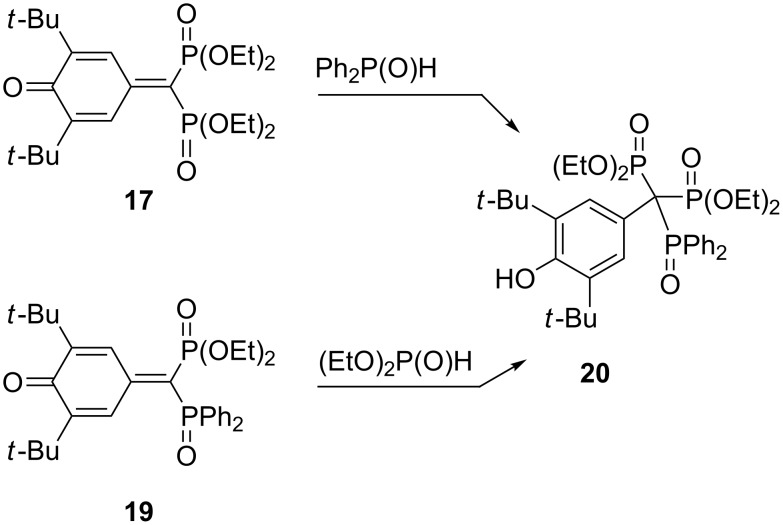
Synthesis of triphosphorus derivatives **20** via quinone methides **17** and **19**.

**Scheme 11:**
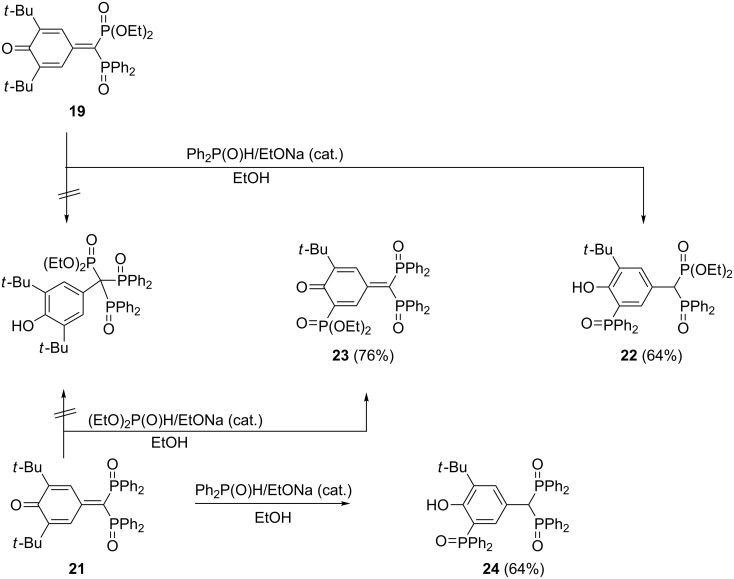
Unexpected phosphonylation of the aromatic nucleus in reactions of quinone methides **19** and **21**.

This reaction mode takes place also for the interactions of quinone methide **17** with diphenylphosphinite and quinone methide **19** with diethyl phosphite (Scheme 10) [33]. However, when the same researchers tried to obtain triphosphorus derivatives with one phosphono and two phosphinoxido groups using quinone methides **19** and **21** as starting materials, phosphonylation of the aromatic nucleus via splitting off a *tert*-butyl group as isobutene and formation of bisphosphonates **22** and **23** was observed. The same happened when quinone methide **21** was treated with diphenylphosphinite (Scheme 11). The primary step of this reaction, which proceeds under mild alkaline conditions, is presumably a direct attack of the diphenylphosphinyl anion in 3-position, followed by the splitting off of isobutene [33–34].

**Scheme 12:**
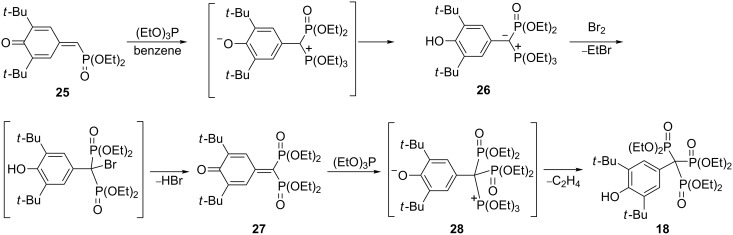
Multistep synthesis of trisphosphonate **18** starting from quinone methide **25**.

Variations on the Arbuzov reaction have also been tested in the preparation of trisphosphonate **18**. Thus, a phosphonylation in three steps could be performed on monophosphonylated quinone methide **25**. The starting compound was first phosphonylated with triethyl phosphite to give the phosphonium betain **26**. This compound was subsequently transformed into the corresponding 7,7-bisphosphonoquinone methide **27** by treatment with bromine. Heating of **27** under reflux in triethyl phosphite resulted in the trisphosphonate **18** in a yield of 40%. The phosphonium betain **28**, which was expected from the reaction of **27** and triethyl phosphite was unstable and could not be isolated (Scheme 12) [35]. It should be noted that compound **18** can be easily oxidized to the corresponding stable phenoxyl radical with PbO_2_ in toluene [36].

#### Synthesis from diazomethylenebisphosphonates

**Scheme 13:**
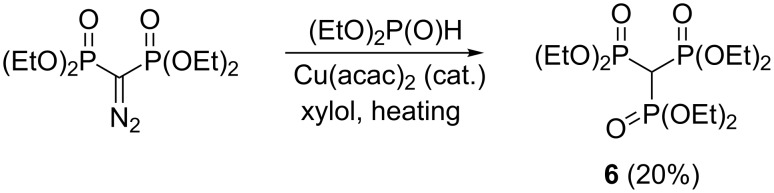
Synthesis of hexaethyl methylidynetrisphosphonate (**6**) via metal-carbenoid-mediated P–H insertion reaction.

The feasibility of synthesizing trisphosphonate esters under mild conditions via metal-carbenoid-mediated P–H insertion reactions was demonstrated by Gross et al. [25]. In particular, the reaction between tetraethyl diazomethylenebisphosphonate and diethyl phosphite in the presence of copper(II) bis(acetylacetonate) provides trisphosphonate ester **6** (Scheme 13). The yield is poor (20%) but the product can be isolated in the pure state and the method is presumably general (cf. [37]). The starting tetraalkyl diazomethylenebisphosphonates are prepared by the reaction of tosyl azide [38–39] or 2-naphthalenesulfonyl azide [40] with the corresponding methylenebisphosphonate precursors in the presence of a base.

#### Various methods

**Scheme 14:**
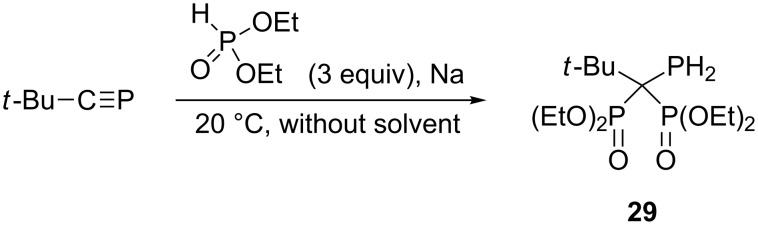
Reaction between *tert*-butylphosphaethyne and diethyl phosphite in the presence of sodium metal.

Quite interestingly, at room temperatures diethyl phosphite reacts with *tert*-butylphosphaethyne in the presence of sodium metal to form 1,1-bis(diethoxyphosphoryl)-2,2-dimethylpropylphosphine (**29**) (Scheme 14) [41]. The proof of structure **29** was given but no details were provided on the reaction course.

### Reactions of methylidynetrisphosphonate esters

**Scheme 15:**
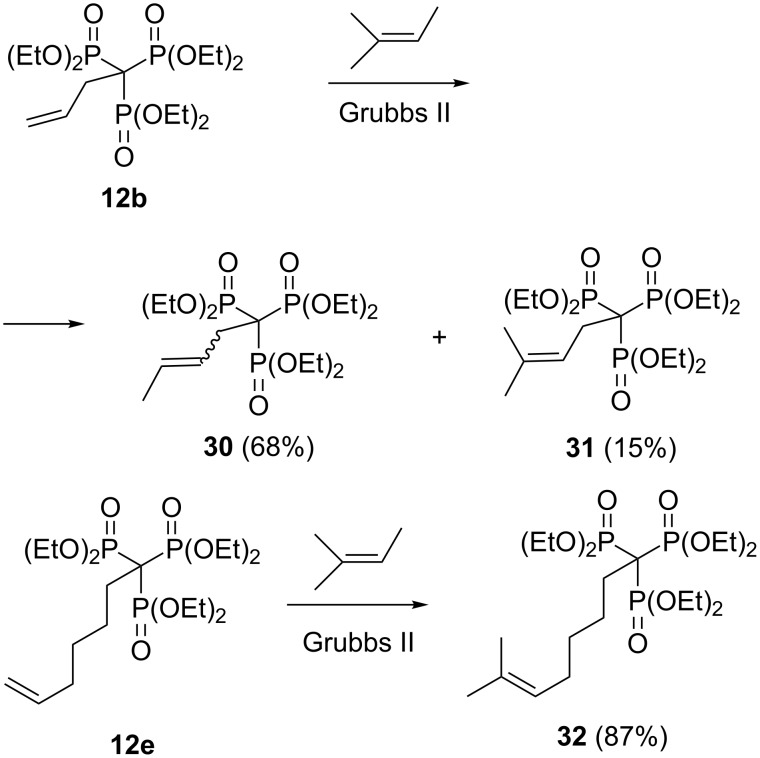
Cross metathesis of trisphosphonates **12** with 2-methyl-2-butene and the Grubbs second-generation catalyst.

The C(PO_3_R_2_)_3_ group is chemically resistant to attack by bases and oxidizing/reducing agents. Upon treatment of hexaethyl methylidynetrisphosphonate (**6**) with NaH in THF, formation of the sodium salt was suggested by a downfield shift in the ^31^P NMR spectrum (from 14 to 32 ppm). However, no further alkylation reaction could be observed with benzyl bromide and allyl bromide, presumably because of high stabilization and strong steric shielding of the carbanionic center [26]. In fact, the importance of steric factors in the reactivity of trisphosphonate esters manifested in many reactions of α-alkyl-substituted trisphosphonates. Thus, attempted cross metathesis of allyl derivative **12b** with 2-methyl-2-butene and the Grubbs second-generation catalyst afforded the unexpected *cis* and *trans-*1,2-disubstituted olefins **30** as the major product and only a small amount of the expected trisubstituted olefin **31**. However, under similar conditions sterically less congested analogue **12e** smoothly undergoes cross metathesis to give the desired trisubstituted olefin **32** in high yield (Scheme 15) [26].

**Scheme 16:**
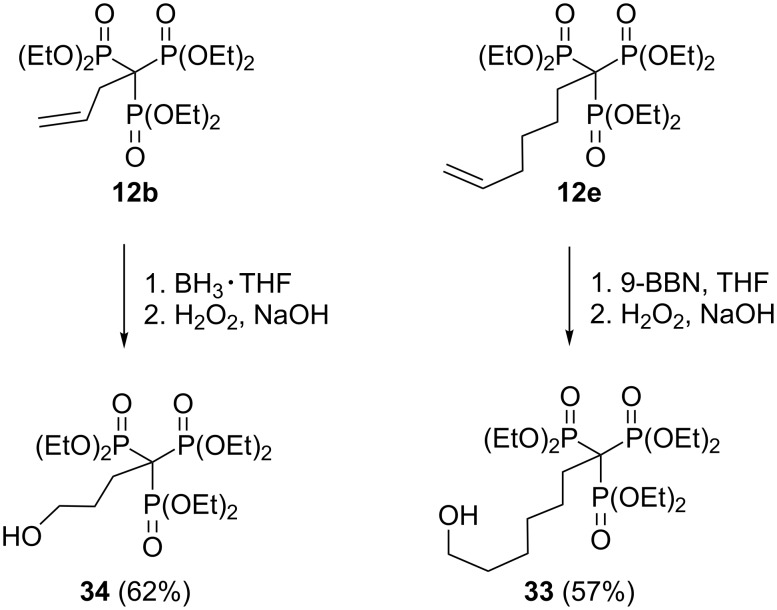
Hydroboration–oxidation of trisphosphonates **12b**,**e**.

**Scheme 17:**
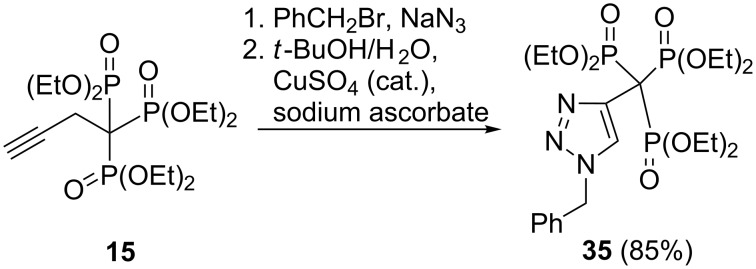
Reaction of 3-butyn-1-ylidenetrisphosphonate **15** with benzyl azide.

In a similar sense, reduction of the trisphosphonate **12e** with 9-borabicyclo[3.3.1]nonane (9-BBN) followed by standard oxidative workup afforded the primary alcohol **33** in reasonable yield, but the 1-allyl-substituted trisphosphonate **12b** did not undergo hydroboration with 9-BBN. However, treatment of **12b** with borane in THF resulted in conversion to the primary alcohol **34** in good yield (Scheme 16) [26]. A further example of the reactivity of trisphosphonates is provided by a click reaction of 3-butyn-1-ylidynetrisphosphonate **15** with benzyl azide, which results in novel triazole compound **35** bearing the trisphosphonate function (Scheme 17) [26].

**Scheme 18:**
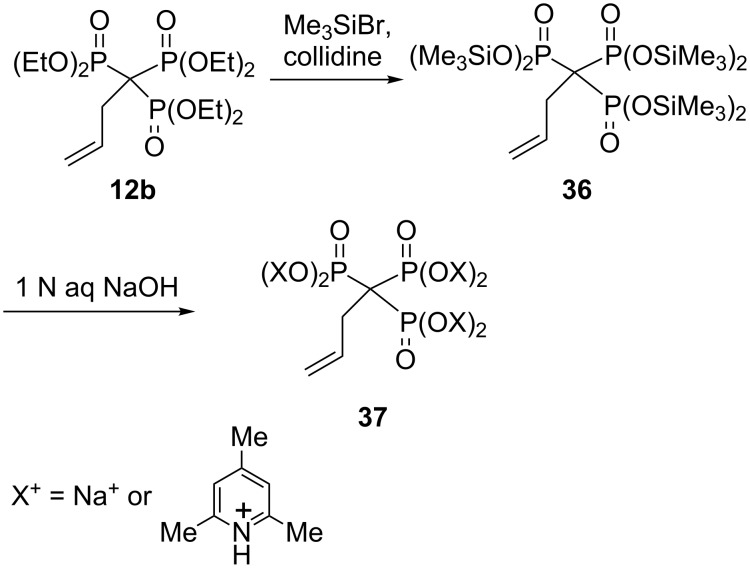
The use of the transsilylation reaction for the synthesis of trisphosphonate salts **37**.

In contrast to methylenebisphosphonate esters, methylidynetrisphosphonate esters have a tendency to undergo dephosphonylation when subjected to acid hydrolysis. Thus, although the bisphosphonate PhCH_2_CH(PO_3_Et_2_)_2_ smoothly undergoes hydrolysis to the corresponding bisphosphonic acid by treatment with HCl under reflux, benzyltrisphosphonate PhCH_2_C(PO_3_Et_2_)_3_ undergoes dephosphonylation under similar conditions [25–26]. Synthesis of free trisphosphonic acids could be carried out by transsilylation of the corresponding hexaalkyl trisphosphonates with Me_3_SiBr in the presence of a base followed by hydrolysis or alcoholysis [42]. This methodology has been particularly successful for preparing the parent methylidynetriphosphonic acid and its salts. Thus, heating **9** with Me_3_SiBr in dichloromethane followed by solvolysis in the presence tri-*n*-butylamine gave methylidynetrisphosphonic acid as its tris(tributylammonium) salt. This product was converted into its trisodium salt by precipitation from a methanol solution using a NaI solution in acetone [7]. The method has been also applied to the preparation of the trisphosphonate salts **37**. Treatment of trisphosphonic acid ester **12b** with Me_3_SiBr and collidine resulted in the formation of the silyl ester **36**, which was converted into a mixed sodium and collidinium salt **37** by the addition of 1 N aqueous NaOH (Scheme 18) [26].

**Scheme 19:**
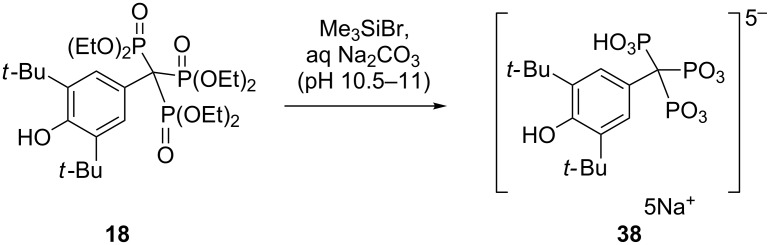
Synthesis of the sodium salt of the acid-labile trisphosphonic acid **38**.

Similarly, a sodium salt of an acid-labile trisphosphonic acid **38** could be prepared with minimal P–C scission by carbonate-buffered hydrolysis of in situ formed silyl ester (Scheme 19) [43].

**Scheme 20:**
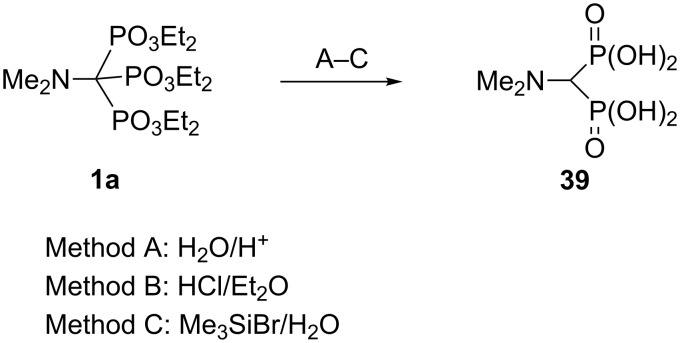
Acidic hydrolysis of trisphosphonate ester **1a**.

Amino-substituted trisphosphonate esters Alk_2_N–C(PO_3_Et_2_)_3_ are even less resistant to acid dephosphonylation than the parent trisphosphonate esters, HC(PO_3_R_2_)_3_, or their α-carbo-substituted derivatives. In particular, in the case of aminotrisphosphonate ester **1a** all standard synthetic routes to phosphonic acids (A–C) via acidic hydrolysis of phosphonate esters lead to the elimination of one phosphonyl group and the formation of bisphosphonic acid **39**. Even with Me_3_SiBr/H_2_O, transsilylation and solvolysis under mild conditions afforded only the dephosphonylated product (Scheme 20) [43].

**Scheme 21:**
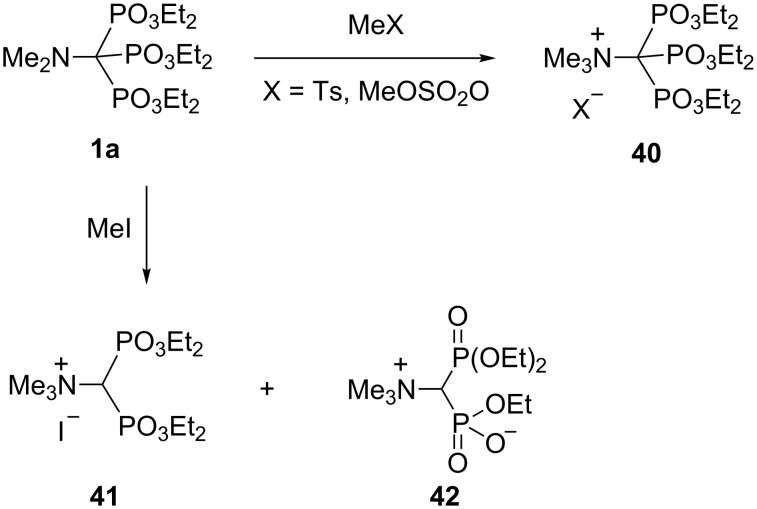
Methylation of trisphosphonate **1a**.

The result of the methylation of trisphosphonate **1a** depends on the reagent: treatment of **1a** with methyl *p*-toluenesulfonate or dimethyl sulfate leads to the expected quarternary ammonium salts **40**, while with iodomethane one phosphoryl group is split off, and a mixture of bisphosphonates **41** and **42** is formed (Scheme 21) [20].

**Scheme 22:**
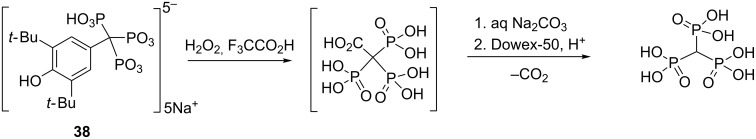
Synthesis of the free methylidynetrisphosphonic acid via trisphosphonate salt **38**.

**Scheme 23:**
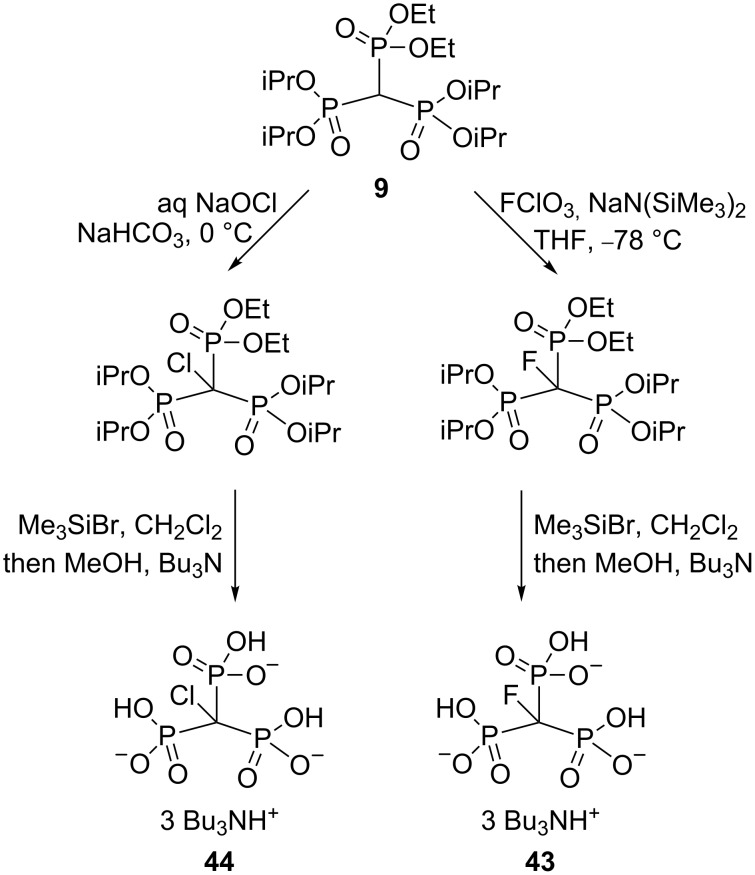
Synthesis of halomethylidynetrisphosphonate salts **43** and **44** by modified Gross’s procedure.

In a fascinating series of publications, Blackburn and co-workers have realized the synthesis of “supercharged” mimics of pyrophosphoric acid capable of introducing additional anionic charge relative to simple methylenebisphosphonates when built into ATP and Ap*_n_*A analogues [7–8,27,44]. They demonstrated that methylidynetrisphosphonic acid and especially its halogenated derivatives are key structure blocks in the synthesis of the nucleotide analogues with enhanced affinity for receptors and better charge correlation with transition states for selected kinases. Two synthetically useful approaches to the parent trisphosphonic acid HC(PO_3_H_2_)_3_ have been developed. One of the procedures is based on the treatment of trisphosphonate salt **38** with a mixture of hydrogen peroxide in trifluoroacetic acid (Scheme 22). An alternative and more efficient synthesis of methylidynetrisphosphonic acid uses a transsilylation of hexaalkyl trisphosphonate **9** followed by hydrolysis [27]. Synthesis of halomethylidynetrisphosphonic acids **43** and **44** is shown in Scheme 23 [7].

**Scheme 24:**
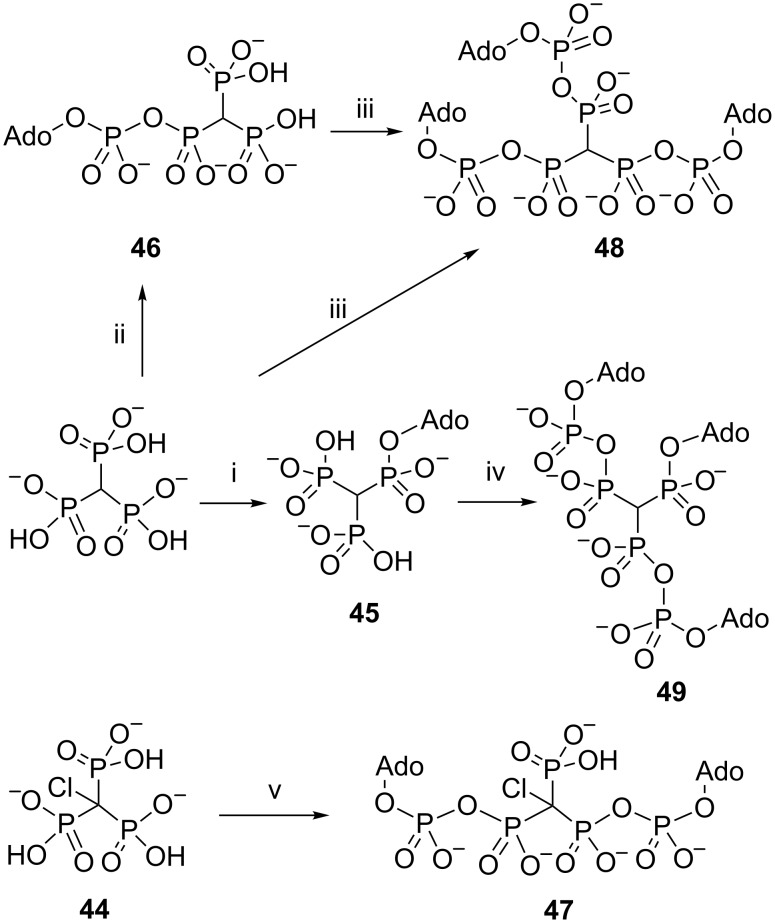
Synthesis of trisphosphonate modified nucleotides. Reagents: i, 5'-*O*-tosyl adenosine, MeCN; ii, AMP–morpholidate (0.8 equiv), tetrazole, pyridine; iii, AMP–morpholidate (5 equiv), tetrazole, pyridine; iv, excess AMP–morpholidate, pyridine; v, AMP–morpholidate (2.2 equiv), tetrazole, pyridine. All counterions are tri-*n*-butylammonium. Ado = 5'-adenosyl.

The tris(tributylammonium) salt of methylidynetrisphosphonic acid was transformed into an ADP analogue **45** and into an analogue of ATP **46** using the method of Poulter and the phosphoromorpholidate procedure of Khorana and Moffatt, respectively. Diadenosine tetraphosphate analogue **47** was obtained upon treatment of chloromethylidynetrisphosphonic acid **44** with excess AMP morpholidate. The incorporation of the third adenylate moiety was found to be extremely slow; however, the use of tetrazole as catalyst allowed the preparation of *P*^1^,*P*^2^,*P*^3^-tris(5'-adenylyl)methylidynetrisphosphonate **48** and the tripodal *P*^1^-5'-adenosyl *P*^2^,*P*^3^-bis(5'-adenylyl)methylidynetrisphosphonate **49** in good yields. Compounds **48** and **49** provide the first examples of species in which three adenylate moieties are linked together by a methylidynetrisphosphonate core (Scheme 24) [8].

### Structure features, spectroscopic and acid properties

Methylidynetrisphosphonic acid as tris(cyclohexylamine) salt, HC(PO_3_H_2_)_3_∙3C_6_H_11_NH_2_, is a stable, easy to handle colorless solid (mp 210–211 °C; ^31^P NMR (H_2_O) δ 12.8 ppm; ^1^H NMR (D_2_O) δ 2.73 (CH), *J*_PCH_ = 22.3 Hz; ^13^C NMR (D_2_O) δ 45.16 (CH), *J*_PC_ = 102.2 Hz) [25]. Methylidynetrisphosphonic acid hexaethyl ester, HC(PO_3_Et_2_)_3_, is a light yellow oil (bp 135–138 °C/0.01 mmHg; ^31^P NMR (CHCl_3_) δ 14.4 ppm; ^1^H NMR (CDCl_3_) δ 3.23 (CH), *J*_PCH_ = 24.2 Hz; ^13^C NMR (CDCl_3_) δ 40.34 (CH), *J*_PC_ = 121.9 Hz) [24–26]. As expected, trisphosphonate ester HC(PO_3_Et_2_)_3_ is a strong carbon acid (titration with NaOH gave a p*K*_a_ of ~6.5) [26].

Under the conception of Blackburn, methylidynetrisphosphonic acid, HC(PO_3_H_2_)_3_, can be viewed as a “supercharged” mimic of pyrophosphoric acid (PP_i_) since the introduction of a third ionizable phosphonate (PO_3_H_2_) group into methylenebisphosphonic acid delivers additional charge at physiological pH. Thus, the parent methylidynetrisphosphonic acid and its fluoro- and chloro-substituted derivatives have at least one more negative charge than pyrophosphate at pH 7 (Table 1) [7,27].

**Table 1:** Ionisation constants for polyphosphonic acids determined in the range 3.5 < pH < 10.5 at 37 °C and 0.152 M NaCl [7].

Polyphosphonic acid	p*K*_a_3	p*K*_a_4	p*K*_a_5	Net charge at pH 7.0

O(PO_3_H_2_)_2_	6.6	9.4	–	2.72
HO_2_CCH(PO_3_H_2_)_2_	–^a^	7.24	10.11	3.35
HO_3_SCH(PO_3_H_2_)_2_	–^a^	6.61	10.57	3.71
HC(PO_3_H_2_)_3_	–^a^	6.46	9.90	3.77
ClC(PO_3_H_2_)_3_	–^a^	5.92	9.08	3.92
FC(PO_3_H_2_)_3_	–^a^	5.77	8.86	3.95

^a^The strongly acidic dissociation constants were off-scale for measurements by titration.

**Figure 1:**
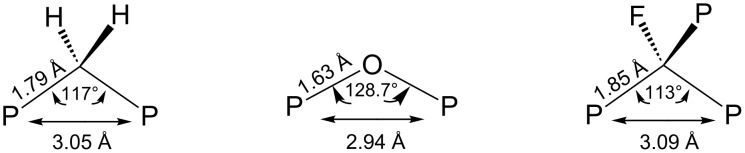
Bond angles and bond distances in pyrophosphate, methylene-1,1-bisphosphonate and fluoromethylidynetrisphosphonate.

X-ray structure analysis of trisphosphonic salt [FC(PO_3_H)_3_]^3−^ 3Na^+^ supported its isosteric character relative to pyrophosphate. In particular, the P–C–P geometry (both the P–C bond distance and the P–C–P angle) is close to the geometry in the methylenebisphosphonate salts while the phosphorus–phosphorus distance is close to that observed for methylenebisphosphonates and pyrophosphate salts (Figure 1) [7].

Structural features of the trisphosphonate **18** were studied by NMR spectroscopy and by single-crystal X-ray diffraction. Only one ^31^P NMR signal is observable for three equivalent phosphonate moieties in CHCl_3_. In contrast, the ^31^P solid-state NMR spectrum of **18** revealed three separable signals. The nonequivalence of the signals was attributed to hydrogen bonds and supported by crystallographic analysis. The molecules **18** bonded via hydrogen bonds form chains [45].

### Short overview of biomedical application

Methylidynetrisphosphonate, HC(PO_3_H_3_)_3_^3−^∙3Bu_3_NH^+^, and its fluoro (**43**) and chloro (**44**) derivatives do not detectably inhibit human-tumor-suppressor protein Fhit, but are strong inhibitors of the lupine enzyme. By contrast, the adenylated polyphosphonates AdoPPCCl(P)PPAdo (**47**) and (AdoPP)_3_CH (**48**) strongly and competitively inhibit Fhit while they are less effective as inhibitors of the lupine enzyme. Since the detection of levels of Fhit protein is an important problem relating to cancers, Fhit-selective inhibitors such as **47** and **48** can be valuable as Fhit diagnostics [8].

β,γ-Chlorophosphonomethylene–ATP, AdoPPCCl(P)P, is a weak antagonist at P2X_2/3_ receptors (IC_50_ about 10 μM) [46].

The antioxidant activity of the compound ArC(PO_3_Et_2_)_3_ (Ar = 2,6-*t*-Bu_2_-4-MeC_6_H_2_) was studied in a model oxidation of oleic acid and with biological objects (liver homogenates of Wistar rats) [36].

Among polyphosphonic acids with a geminal arrangement of phosphonic groups efficient complexones and regulators of calcium exchange in humans were found [47–49]. Some data on the use of the methylidynetrisphosphonic acid and its derivatives as complexones were also published. The trisphosphonic acids HC(PO_3_H_2_)_3_ and ClC(PO_3_H_2_)_3_ are better chelating agents in the detergent compositions than methylenebisphosphonic acid and its alkali-metal salts and also sequester more calcium and magnesium ions, for example, than does H_2_C(PO_3_H_2_)_2_ [50–51]. The complexation behavior of the polydentate ligand Me_2_NC(PO_3_Et_2_)_3_ toward Co^2+^ ion has shown that the trisphosphonate molecule is coordinated in solution by its three donor (P=O, Me_2_N) functions [52]. Evidently, further detailed structure studies of the individual complexes and the complex-forming driving factors are desired in order to understand trisphosphonate coordination abilities.

## Conclusion

There has been a considerable interest in the preparation and use of the geminal trisphosphonates, XC(PO_3_R_2_)_3_, because of the widespread biomedical application of methylenebisphosphonates as mimetics of biologically important pyrophosphate. Much of the trisphosphonate reactivity profile follows intuition based on the bisphosphonate analogy. However, despite the structural similarity to bisphosphonates, methylidynetrisphosphonic acid and its derivatives differ in their geometry, coordination properties and reactivity pathways. A particularly interesting characteristic of trisphosphonates is the possibility of constructing systems based on Blackburn’s conception of supercharged nucleotide analogues in which an additional negative charge is provided without elongation of the polyphosphate chain. But there are still a lot of other aspects of their chemistry that remain to be investigated. From a synthetic point of view, since the introduction of a heteroatom substituent at the bridged carbon atom permits both modulation of p*K*_a_ values and hydrogen bonding, there is a need for profound study of α-functionalized trisphosphonate systems. Such compounds can be promising building blocks for the synthesis of false substrates or enzyme inhibitors involved in phosphate-based processes. In particular, some heterocyclic compounds functionalized by trisphosphonate substituents merit in-depth biological study. A further practical potential for trisphosphonate compounds is the development of new phosphorus-containing dendrimers and related species. Considerable interest is also associated with the use of trisphosphonic acids as ligands for calcium ligation and as potential bone affinity agents. Finally, there is no doubt that organometallic and coordination chemistry will benefit from future innovative application of these compounds.
